# Periodontitis-Derived Dark-NETs in Severe Covid-19

**DOI:** 10.3389/fimmu.2022.872695

**Published:** 2022-04-12

**Authors:** Ljubomir Vitkov, Jasmin Knopf, Jelena Krunić, Christine Schauer, Janina Schoen, Bernd Minnich, Matthias Hannig, Martin Herrmann

**Affiliations:** ^1^ Clinic of Operative Dentistry, Periodontology and Preventive Dentistry, Saarland University, Homburg, Germany; ^2^ Department of Environment & Biodiversity, University of Salzburg, Salzburg, Austria; ^3^ Department of Dental Pathology, University of East Sarajevo, East Sarajevo, Bosnia and Herzegovina; ^4^ Department of Internal Medicine 3 - Rheumatology and Immunology, Friedrich-Alexander-University Erlangen-Nürnberg (FAU) and Universitätsklinikum Erlangen, Erlangen, Germany; ^5^ Deutsches Zentrum für Immuntherapie (DZI), Friedrich-Alexander-University Erlangen-Nürnberg and Universitätsklinikum Erlangen, Erlangen, Germany

**Keywords:** neutrophil hyper-responsiveness, dysregulated immunity, trained immunity, NET hyper-responsiveness, NET-induced damage, inhibition of NET formation

## Abstract

The frequent severe COVID-19 course in patients with periodontitis suggests a link of the aetiopathogenesis of both diseases. The formation of intravascular neutrophil extracellular traps (NETs) is crucial to the pathogenesis of severe COVID-19. Periodontitis is characterised by an increased level of circulating NETs, a propensity for increased NET formation, delayed NET clearance and low-grade endotoxemia (LGE). The latter has an enormous impact on innate immunity and susceptibility to infection with SARS-CoV-2. LPS binds the SARS-CoV-2 spike protein and this complex, which is more active than unbound LPS, precipitates massive NET formation. Thus, circulating NET formation is the common denominator in both COVID-19 and periodontitis and other diseases with low-grade endotoxemia like diabetes, obesity and cardiovascular diseases (CVD) also increase the risk to develop severe COVID-19. Here we discuss the role of propensity for increased NET formation, DNase I deficiency and low-grade endotoxaemia in periodontitis as aggravating factors for the severe course of COVID-19 and possible strategies for the diminution of increased levels of circulating periodontitis-derived NETs in COVID-19 with periodontitis comorbidity.

## Introduction

Periodontitis has been considered not a consequence of basic alteration of the oral microbiota, but rather of the inability of the host immunity to resolve chronic inflammation (1–3). Indeed, the capacity of certain bacteria to act as a commensal or pathogen is highly dependent on the immune status of the host (4). Periodontitis patients with late onset have low-grade endotoxaemia (LGE), systemic low-grade inflammation (SLGI) (5) and display neutrophil hyper-responsiveness (6–9). Neutrophils play a central role in the control of bacterial infections and also are the effector immune cells responsible for the antimicrobial defence in the gingiva. They are the first defenders recruited to the sites of bacterial invasion (1, 2, 10). The indispensable role of neutrophils is illustrated by the inevitable development of early-onset periodontitis in patients with neutropenia and with congenital defects of leukocyte adhesion (1). Neutrophils do not recognise individual pathogens or pathogen species, but common danger signals such as: chemokines, cytokines, immune complexes, pathogen-associated molecular patterns (PAMPs), damage-associated molecular patterns (DAMPs), and certain proteins of the complement system (11). PMN hyper-reactivity is characteristic of periodontitis with late onset and can evoke a strong inflammatory response even at low levels of bacterial challenge. Consequently, neutrophils can transform from defenders against pathogens to tissue destroyers regardless of the bacterial challenge (11, 12).

NETs are part of the antimicrobial arsenal of neutrophils (13–17). They are evolutionarily conserved chromatin threads produced by activated neutrophils in response to pathogen challenge. NETs consist of a scaffold of chromatin with histones and neutrophil-derived antimicrobials, such as: proteases, lactoferrin, cathepsins and myeloperoxidase (MPO) (13, 14, 18). NET formation can be triggered *via* receptors sensing chemokines and cytokines, immune complexes, PAMPs, DAMPs, some complement components (19–21), *via* pH regulation (22), and *via* activating the caspase-4/5/GSDMD pathways (23). Human caspases 4 and 5 are receptors for cytosolic LPS (24). Many of the mechanisms for NET formation are linked to the NADPH oxidase (NOX) machinery, but NOX-independent NET formation has also been described (23, 25). Upon activation, the azurophilic granular proteins neutrophil elastase (NE) and MPO translocate to the nucleus to promote chromatin decondensation (26). Histone citrullination by peptidylarginine deiminase 4 (PAD4) further supports this (27, 28). In addition, reactive oxygen species- (ROS), NE- and PAD4-independent pathways for NET formation have been reported. Such as the caspase-4/11-induced NET formation that proceeds independently of MPO, NE, and PAD4 (23).

Circulating NETs can exert strong proinflammatory effects. NETs induce the inflammasome (29), type I interferons and further pro-inflammatory cytokines; they damage the endothelium (30) and can occlude ducts in various organs, promoting organ damage (31–39). In patients with COVID-19 aggregated NETs (aggNETs), can obstruct small and intermediate-sized pulmonary vessels and precipitate COVID-19 pathology (40). Recently, an association between periodontal disease and an increased risk of COVID-19 infection has been reported (41). Periodontitis was associated with a higher risk of intensive care unit (ICU) admission, need for assisted ventilation and death of patients with COVID-19 (41–43). These findings identify periodontitis as comorbidity that drives COVID-19. Both COVID-19 (44–46) and periodontitis (5) share a dysregulated innate immunity.

The aim of this review is to discuss the disbalanced NET formation in periodontitis, especially in the context of COVID-19. Furthermore, we want to highlight possible approaches to counteract the increased level of NETs reported in periodontitis with late onset.

## NETs in Periodontitis With and Without COVID-19 Comorbidity

### Sources of Circulating NETs in COVID-19 and Periodontitis

NETs in blood can be directly demonstrated by vascular biopsies (40), but this approach is inapplicable for clinical purposes. Blood-born NETs are physiologically digested by DNase 1 into cell-free DNA (cfDNA). The main source of blood-born cfDNA originates from cell necrosis and apoptosis, as cases of neoplasms are, as well as NETs (47). Thus, patients with severe COVID-19 had an increased level of nuclear cfDNA (copies/mL), as compared to hospitalised non-ICU COVID-19 patients and the cfDNA sequencing identified the neutrophils as a predominant cfDNA source (48). Clinically, cfDNA is highlighted as a non-invasive biomarker for COVID-19 severity (49). Additionally, topical formation of NETs, as the case with periodontitis is (50–52) and neutrophil hyper-responsiveness both in periodontitis (6–9) and COVID-19 (53) are strong indicators of blood-born NETs. It has been demonstrated that the circulating carbamylated protein (54, 55) and NET levels are associated with periodontitis severity (56). Blood cfDNA is significantly increased in patients with periodontitis, as compared to orally healthy subjects (57). Thus in periodontitis, topical sources of NETs, neutrophil hyper-responsiveness and circulating NETs alter the host reactivity in the same way as in severe COVID-19 and provide the requirements for aggravation of NET-driven host damages.

### Increased NET Formation Due to Trained Immunity

Most proinflammatory gene loci in the quiescent myeloid cells are in a repressed configuration (58), hindering access of the transcriptional machinery to the regulatory regions that drive the expression of inflammatory factors (59). The so-termed “trained immunity” is development of a long-term functional reprogramming of the hematopoietic stem and progenitor cells (HSPCs) evoked by exogenous or endogenous insults, e.g. low level LPS due to LGE, as the case in periodontitis with late onset is. Subsequently, this functional reprogramming of HSPCs leads to an altered responsiveness of differentiated myeloid cells towards a second challenge after returning to a non-activated state (60). The secondary response to the subsequent non-specific stimulus can be altered in such a way that the cells respond more or less strongly when compared to the primary response, conferring context- and time-adjusted responses (60). Thus, low level LPS-exposed HSPCs become epigenetically primed for a myeloid lineage bias with enhancers remaining more accessible than in naive HSPCs. Furthermore, low level LPS-exposed HSPCs keep increased accessibility of numerous genes that predispose to more rapid activation of myeloid lineage commitment in response to secondary stimulation (61). As the innate memory responses depend solely on epigenetic remodelling, the trained immunity lacks specificity. Trained neutrophils are prone to increased NET formation (62, 63). Continuous LGE promotes systemic low-grade inflammation (SLGI) and subsequently dysregulated trained immunity in periodontitis with late onset (3, 5, 64). Thus, the NET hyper-responsiveness might be a main factor for increased NET formation in periodontitis patients and hence responsible for a more severe course of COVID-19 with periodontitis comorbidity (41, 42).

### Impaired DNA Degradation

The disrupted balance between NET formation and degradation appears to play a crucial role in the pathophysiology of inflammation, coagulopathy, organ damage and immunothrombosis that characterise severe cases of COVID-19 (45). In general, impaired NET degradation results in surplus of NETs causing a multitude of tissue damages. Thus, delayed NET degradation in systemic lupus erythematosus plays a crucial role in the lupus pathology (65, 66). NETs have been shown to initiate several detrimental effects directly on the host (45), especially when the NET degradation is impaired (67), as the case in COVID-19 is (68). One of the mechanisms responsible for delayed NET degradation is surplus of DNase I inhibitors (65), foremost G-actin, which forms a complex with DNase I, thereby inhibiting its nuclease activity (69). LPS signalling induces reorganisation of cell microfilaments and after LPS stimulation a subsequent rapid actin disassembly occurs (70). LPS increases the cellular G-actin pool without a reciprocal decrease in the F-actin pool. The G-actin increment could be explained, in part by F-actin depolymerisation, and in part, by *de novo* actin synthesis. This new actin synthesis could be a cell response to LPS challenge to maintain actin cytoskeletal integrity and barrier function. That LPS stimulates actin synthesis has been further substantiated by an increased total actin pool (71). In humans, G-actin levels were greatest in systemic inflammatory syndromes, but significantly elevated in septic shock as compared with healthy subjects (72). Similarly, delayed NET degradation in patients with periodontitis has been reported (73, 74). As periodontitis with late onset is paired with low-grade endotoxaemia (5), the connection between endotoxaemia and increased G-actin level respectively delayed NET degradation, suggests itself. Periodontitis treatment decreases endotoxemia and hence attenuates the delayed NET degradation (74).

### Endotoxemia in COVID-19 Boosts NET Formation

**Figure 1:**
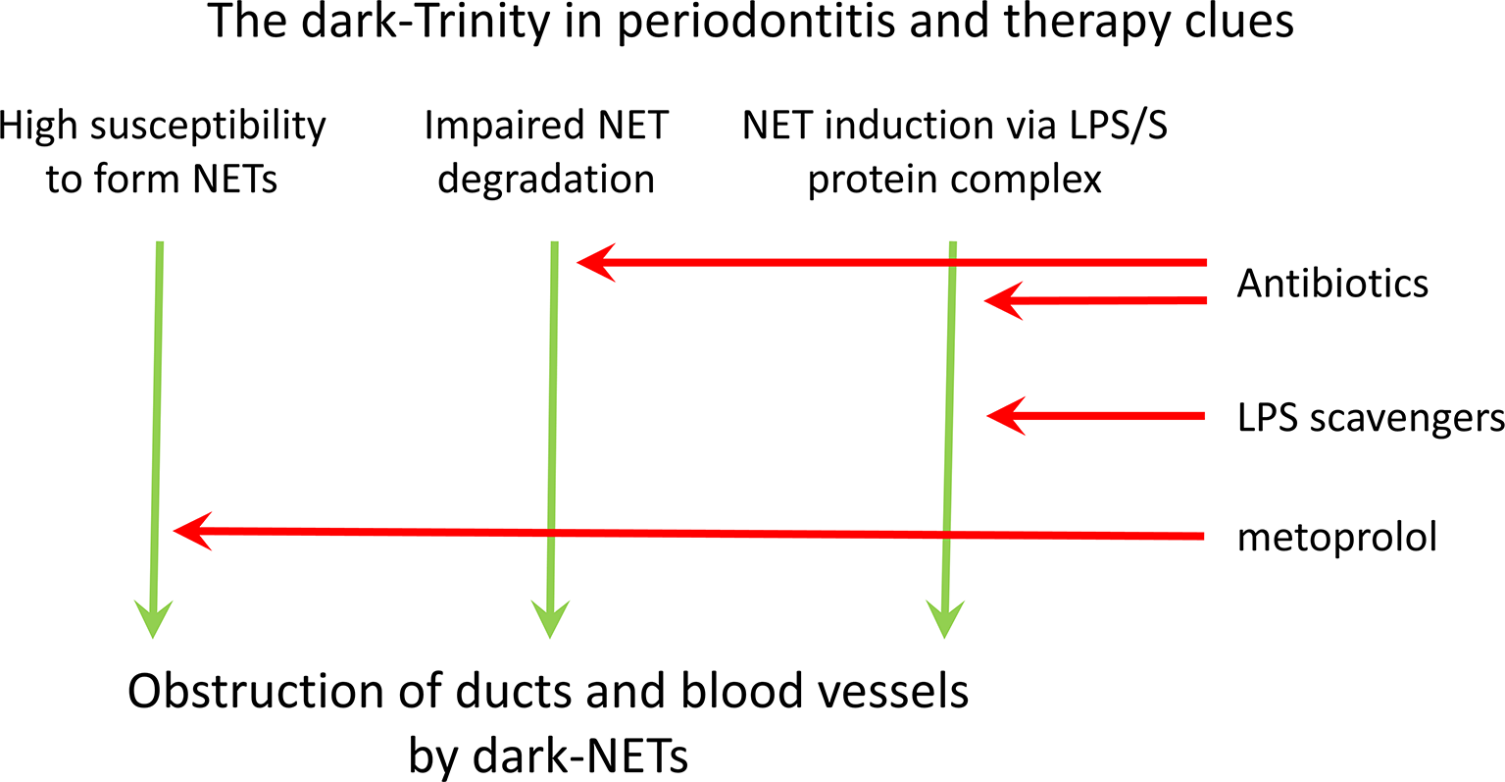
Pathogenic factors and treatment approaches in severe COVID-19 course in patients with periodontitis.

LGE, i.e. increased plasma levels of LPS and LPS-binding protein (LBP), is associated with obesity (75, 76), diabetes (75–77), cardiovascular diseases (CVD) (78, 79), gut microbiome dysbiosis (80), and periodontitis (81–84). Although the mechanisms responsible for LGE in these diseases differ, they are all characterised by increased LPS blood serum levels. In periodontitis, LPS serum level is in the range of 0.89 ± 2.90 ng/ml (85). SARS-CoV-2 binds to bacteria or directly to free LPS, thereby enhancing their attachment to ACE2 receptors on the host cell surface. SARS-CoV-2 directly interacts with LPS through its S protein (86). Neither SARS-CoV-2 spike protein (S protein) nor LPS alone causes any activation of the pro-inflammatory nuclear factor kappa B (NF-κB), but the combination of S protein and low level of LPS activates NF-κB in a dose-dependent manner (86). The SARS-CoV-2/LPS interactions dramatically increase the viral infectivity and promote the development of hyper-cytokinaemia (75, 87). This mechanism may explain why above mentioned cases of LGE are associated with severe COVID-19 comorbidity (75, 88). **Figure 1** demonstrates three main mechanisms responsible for the emergence of blood-born NETs in periodontitis with and without COVID-19 comorbidity.

## Pathogenic Effects of NETsin COVID-19

Most individuals infected with SARS-CoV-2 remain asymptomatic, luckily just a few severe cases transit into acute respiratory distress syndrome (ARDS), multi-organ failure, and even death (89). Individuals that develop severe manifestations of COVID-19 have signs of dysregulated innate and adaptive immune responses (45). Three main COVID-19 traits have to be considered: (I) dysregulated immune responses, (II) surplus of circulating NETs and (III) NET-mediated damage of distant organs *via* immunothrombosis (45). Nevertheless, many different mechanisms are responsible for excessive NET triggering in COVID-19:

SARS-CoV-2 activates complement regulators and complement (90), which augments the cytokine storm and coagulopathy, two dangerous complications in severe COVID-19 (90). Complement activation also triggers NETs in COVID-19 (19).

Sera from COVID-19 patients triggers NET release by healthy control neutrophils *in vitro* (91). Indeed, viable SARS-CoV-2 directly stimulate human neutrophils to release NETs in a dose-dependent manner. Neutrophil-expressed ACE2, TMPRSS2, and viral replication are generally required for this SARS-CoV-2-induced NET formation (92).

The conjunction of LPS and SARS-CoV-2 S protein activates toll-like receptor 4 (TLR4) in extremely low concentrations of LPS, e.g. of 1ng/ml, causing NET formation *via* the NF-κB activation (86), is a phenomenon playing a crucial role for the severe COVID-19 course in patients with LGE such as those with obesity, diabetes, CVD and periodontitis. In addition, LGE in patients with COVID-19 is associated with thrombotic events, possibly due to activation of platelet TLR4 (93). SARS-CoV-2- respectively SARS-CoV-2/LPS-activated platelets form aggregates with leukocytes, in particular in patients with severe disease (40, 94–98). Platelets adhere to injured blood vessels, become activated, and subsequently express adhesion molecules, such as P-selectin and ICAM-1, which induce neutrophil recruitment (45). Platelets are major instigators of direct neutrophil activation (99, 100). Circulating platelets bind neutrophils only in cases of bacterial (101) or viral infection (102) by means of integrins (103). Platelet activation may trigger the formation of intravascular NET aggregates in the pulmonary and renal microcirculation (40, 104) aggravating the course of COVID-19 (105).

## Moderation of Circulating NETs in Periodontitis Patients With Covid-19 Comorbidity

### Moderating the NET Formation

The main complication in severe COVID-19 appears to be the NET-driven ARDS and thrombosis of pulmonary vessels (40, 45). For that reason, anti-NET therapies should target either acceleration of NET degradation (68) or suppression of NET formation (106).

In general, attenuation of topical periodontal inflammatory events does not alter the systemic neutrophil responsiveness, as an enhanced neutrophil responsiveness persists even years after loss of all teeth. This is probably due to the trained immunity (5). The dysregulated trained immunity is responsible for surplus of circulating NETs in periodontitis (2) and relies on epigenetic alteration of HSPCs (3, 5). HSPCs chronically exposed to low level LPS, e.g. as a consequence of periodontitis, become epigenetically primed for a myeloid lineage bias with enhancers remaining more accessible than in naive HSPCs. Furthermore, LPS-exposed HSPCs keep increased accessibility of numerous genes that predispose to more rapid activation of myeloid lineage commitment in response to secondary stimulation than in naive HSPCs (107). This pre-programed chromatin accessibility for other transcription factors, facilitates the response of DNA regulatory elements to stimulation (108). The molecular basis of the epigenetic modifications includes changes in chromatin organization at the level of the topologically associated domains, transcription of long non-coding RNAs, methylation and acetylation of genes involved in the innate immune responses and reprogramming of cellular metabolism (60). Attenuating the neutrophil hyper-responsiveness due to dysregulated trained immunity (109) is an attractive option to ameliorate the course of NET-induced pathologies. Accordingly, many possibilities come into consideration. Two key epigenetic marks characterise trained immunity: (I) the acetylation of histone 3 lysine 27 (H3K27ac) at distal enhancers (marked by histone 3 lysine 4 methylation (H3K4me1) and (II) the consolidation of histone 3 lysine 4 trimethylation (H3K4me3) at the promoters of stimulated genes (60). Histone deacetylase (HDAC) inhibitors appear at first glance to be suitable candidates for this purpose, as they silence the inflammatory genes (110), which became accessible *via* acetylation of histone 3 lysine 27 (60). Indeed, HDAC inhibitors efficiently suppress NET formation (62, 63, 111, 112). However, due to severe side effects, they have not yet been used in humans except for cancer therapies.

PAD4 inhibitor GSK484, employed to suppress NET-induced gallstone blocks, completely overturned exaggerated NET formation and prevented gallstone formation in a murine model (31). This indicates the possibility to use PAD4 inhibitors to ameliorate NET-induced pathology. GSK484 is currently used in clinical studies.

The beta-1 blocker metoprolol reportedly stunned neutrophils (113) and suppressed exaggerated NET formation in mice (31). Of all tested beta-blockers, only metoprolol significantly attenuated exacerbated inflammation and reduced neutrophil infiltration and their interactions with other cell types (114). Recently, metoprolol has been employed for the treatment of ARDS in COVID-19. Its administration was safe and lacked serious side effects (106). Administration of beta-blockers is generally safe except for patients with acute pump failure. Metoprolol has also reduced NET levels and other markers of pulmonary inflammation as well as improved oxygenation (106). Metoprolol-treated patients spent fewer days on invasive mechanical ventilation. The use of metoprolol to treat COVID-19–associated ARDS appears to be a safe and inexpensive strategy that can alleviate the burden of the COVID-19 pandemic.

### Attenuating the Delayed NET Degradation and Endotoxemia Decreasing

NET degradation in periodontitis is reduced (73, 74), as compared to healthy subjects. DNase I is inhibited by G-actin, which (69), which is LGE-induced (71). Delayed NET degradation in LGE contributes to severe COVID-19 comorbidity (88). In addition, the conjunction of low-level LPS and SARS-CoV-2 S protein results in excessive NET formation (86), a phenomenon playing a crucial role for the severe COVID-19 course in patients with periodontitis. Both LPS effects on the host cause NET increase, hence a reduction of LPS level is needed. The antibiotic treatment is a possible adjunctive option both to attenuate LGE effects and the delayed NET degradation in periodontitis with the severe COVID-19. Endotoxin adsorbent therapy in severe COVID-19 pneumonia has been applied, but despite some amelioration, the clinical benefit remains unclear (115). In contrast, antibiotic therapy is uncomplicated and has few or no side effects. Gut microbiota treatment with broad-spectrum antibiotics reduces metabolic endotoxaemia in patients with type 2 diabetes by reduction of LPS production in Gram-negative bacteria (116). Also antibiotic treatment in periodontitis cases effective reduction of blood serum LPS (117) In contrast, dental surgery and oral hygiene manipulations in patients with the severe COVID-19 have to be avoided, as they induce bacteraemia. Other possibilities to reduce LGE and to restore the impaired NET degradation are beyond the scope of periodontitis-related pathology and treatment. **Figure 1** demonstrates the treatment approaches for attenuation of NET surplus in periodontitis with COVID-19 comorbidity.

## Conclusions

Periodontitis with late onset is concomitant with side effects such as neutrophil hyper-responsiveness, propensity to NET formation, circulating NETs and cfDNA, low-grade endotoxaemia and delayed NET degradation. LPS effectuates delayed NET degradation *via* DNase I inhibition and binds the SARS-CoV-2 S protein causing NET production *via* NF-κB activation. These immunity alterations appear to contribute to the severe COVID-19 course in patients with periodontitis, as they cause surplus of NETs. In the viscera and in particular within blood vessels, the DNA scaffold of exaggerated NETs causes obstruction of whole organs and blood coagulation, resulting in heavy damages or even death. No canonical therapy for mastering the exaggerated NET formation has been established to date. The only candidate, promising to ameliorate neutrophil and NET hyper-responsiveness with petty side effects if any, is the beta-1 blocker metoprolol, but further investigations are needed. Adjunctive antibiotic treatment may reduce the periodontitis-relied LGE.

## Author Contributions

Conceptualization and writing— preparation of the original draft, LV. Writing—review and editing: CS, JS, JKn, JKr, LV, BM, MHe, and MHa. All authors have read and agreed to the published version of the manuscript.

## Funding

This research was supported by the German Research Foundation (DFG) Grants No. 2886 PANDORA B3; SCHA 2040/1-1; CRC1181(C03); TRR241(B04), by the EU H2020-FETOPEN-2018-2019-2020-01; 861878 ”NeutroCure”, and by the Volkswagen-Stiftung (Grant 97744).

## Conflict of Interest

The authors declare that the research was conducted in the absence of any commercial or financial relationships that could be construed as a potential conflict of interest.

## Publisher’s Note

All claims expressed in this article are solely those of the authors and do not necessarily represent those of their affiliated organizations, or those of the publisher, the editors and the reviewers. Any product that may be evaluated in this article, or claim that may be made by its manufacturer, is not guaranteed or endorsed by the publisher.

## Abbreviations

ACE2, Angiotensin-converting enzyme 2; ARDS, acute respiratory distress syndrome; cfDNA, cell-free DNA; CVD, cardiovascular diseases; GCF, gingival crevicular fluid; HDAC, histone deacetylase; HSPCs, hematopoietic stem and progenitor cells; ICU, intensive care unit; IL, Interleukin; LBP, LPS-binding protein; LGE, low-grade endotoxemia; LPS, lipopolysaccharide; MMP, matrix metalloprotease; MPO, myeloperoxidase; NE, neutrophil elastase; NETs, neutrophil extracellular traps; NF-κB, nuclear factor kappa-light-chain-enhancer of activated B cells; NOX, NADPH oxidase; PAD4, peptidylarginine deiminases 4; PAMPs, pathogen associated molecular patterns; ROS, reactive oxygen species; SLGI, systemic low-grade inflammation; S protein, spike protein of SARS-CoV-2; TLR4, toll-like receptor 4; TMPRSS2, transmembrane serine protease 2; Treg, Regulatory T cells.
